# MS treatment trends before, during, and after the COVID-19 pandemic: insights from the German MS Register

**DOI:** 10.1007/s00415-025-13010-6

**Published:** 2025-03-26

**Authors:** Agni-Maria Konitsioti, Sarah Laurent, David Ellenberger, Alexander Stahmann, Paulus Rommer, Judith Haas, Clemens Warnke

**Affiliations:** 1 Department of Neurology, Medical Faculty, University Hospital of Cologne, Kerpener Str. 62, Cologne, Germany; 2 Neurology, CHU Saint-Pierre, Université Libre de Bruxelles, Brussels, Belgium; 3 German MS-Register, MS Forschungs-und Projektentwicklungs gGmbH (MS Research and Project Development gGmbH [MSFP]), Hannover, Germany; 4 Department of Neurology, Medical University of Vienna, Vienna, Austria; 5 Neuroimmunological Section, Department of Neurology, University of Rostock, Rostock, Germany; 6 Deutsche Multiple Sklerose Gesellschaft, Bundesverband e.V. (German Multiple Sclerosis Society [DMSG]), Hannover, Germany; 7 Department of Neurology, University Hospital Marburg, Marburg, Germany

**Keywords:** Disease modifying therapy, Risk, Benefit, Pandemic

## Abstract

**Background:**

The COVID-19 pandemic affected healthcare management for people with multiple sclerosis (PwMS), leading to alterations in disease-modifying therapies (DMTs) due to concerns about COVID-19 outcomes and vaccine efficacy.

**Objectives:**

To compare DMT prescription patterns in PwMS before, during, and after the COVID-19 pandemic.

**Methods:**

PwMS from the German MS Register, between 2019 and 2024, either newly diagnosed (Cohort A) or who discontinued or switched DMT (Cohort B), were analyzed over a follow-up period of 3 months. Data from the pre-pandemic period were compared to early-, late-, and post-pandemic periods. DMTs were categorized as medium efficacy (meDMT) or high efficacy (heDMT).

**Results:**

In Cohort A (*n* = 1810), pre-pandemic 46% had no DMT within 3 months of diagnosis, 39% received meDMT, and 15% heDMT (7.5% B cell-depleting therapies (BCD)). heDMT use increased during later periods (“early” 19%, “late” 29%, “post” 41%), with a shift toward BCD. In cohort B (*n* = 4246), pre-pandemic 47% paused DMT, 19% switched to meDMT, and 34% to heDMT (17% BCD). heDMT use also rose during the pandemic (“early” 37%, “late” 47%, “post” 48%), with increased BCD use.

**Conclusions:**

There were no delays in DMT initiation or resumption during the pandemic with a notable increase in heDMT and BCD use, reflecting growing confidence in these treatment options.

## Introduction

The global spread of coronavirus disease-19 (COVID-19) caused by SARS-CoV-2 had a profound impact on healthcare systems worldwide, including the management of people with multiple sclerosis (PwMS) [1]. Numerous national and international registries were adapted to monitor COVID-19 outcomes and disease-modifying therapy (DMT) use in PwMS [2–5]. In March 2020, the European Committee for Treatment and Research in Multiple Sclerosis (ECTRIMS) launched a global survey among MS specialists, revealing, along with similar results from other reports [4], significant disruptions in MS care, including challenges in accessing magnetic resonance imaging (MRI) services, laboratory monitoring, and clinical practice adjustments [6]. Patient surveys echoed these findings, reporting difficulties in maintaining care, DMT discontinuations, and treatment modifications due to COVID-19 fears [7–9].

Risk factors for COVID-19 severity in PwMS are consistent with those in the general population, including older age, male sex, higher disability levels, progressive MS course, obesity, and comorbidities [2, 3, 10–13]. While DMTs have not been consistently identified as significant risk factors for severe COVID-19 outcomes [2, 12, 14–17], there remain concerns regarding the use of immunosuppressive therapies, particularly anti-CD20 monoclonal antibodies and possibly also sphingosine-1-phosphate (S1P) modulators.

For example, the ECTRIMS survey identified severe COVID-19 cases and fatalities predominantly among PwMS receiving DMTs [2, 6], although other studies reported mild to moderate outcomes [18].

As a result, the pandemic has led to notable shifts in DMT-prescribing patterns for PwMS [19, 20]. Interferons and glatiramer acetate were generally prescribed without major concerns [6, 21, 22]. Likewise, natalizumab has not been associated with worse COVID-19 outcomes and may even offer protective effects by limiting viral entry into cells through integrin blockade [25]. PwMS treated with cladribine were generally considered to mount effective immune responses and experience mild COVID-19 symptoms, at least if not infected closely to the last treatment cycle [23, 24]. Accordingly, a multicenter study involving 8,771 patients reported a preference for natalizumab and cladribine over anti-CD20 mAbs and fingolimod, likely to maintain therapeutic efficacy while mitigating immunosuppressive risks during the pandemic [26]. However, there was a marked reduction in the initiation of or escalation to other higher-efficacy disease-modifying therapies, such as alemtuzumab, anti-CD20 monoclonal antibodies (mAbs), and S1P modulators [19, 21, 22, 27].

Retrospective studies suggest that prolonged anti-CD20 therapy may increase the risk of severe COVID-19 in PwMS [13, 17, 18, 28, 29]. Similarly, rituximab has been associated with more severe COVID-19 outcomes in both PwMS and individuals with rheumatic diseases [14, 30, 31].

Concerns both about a more severe COVID-19 disease course during certain DMT and reduced protection following COVID-19 vaccination have significantly challenged MS care, leading to various consensus agreements and recommendations [32, 33]. The prevailing consensus is that the benefits of ongoing MS treatment generally outweigh the risks of discontinuation [15]. To minimize severe infection risks while maintaining disease control, modifications like extended interval dosing were temporarily suggested for heDMT, particularly in B cell-depleted or hypogammaglobulinemic patients [15, 33, 34]. Given the diverse risks associated with each DMT, treatment decisions should be tailored to individual patient needs rather than applying a uniform approach.

## Materials and methods

### Study design

**Fig. 1:**
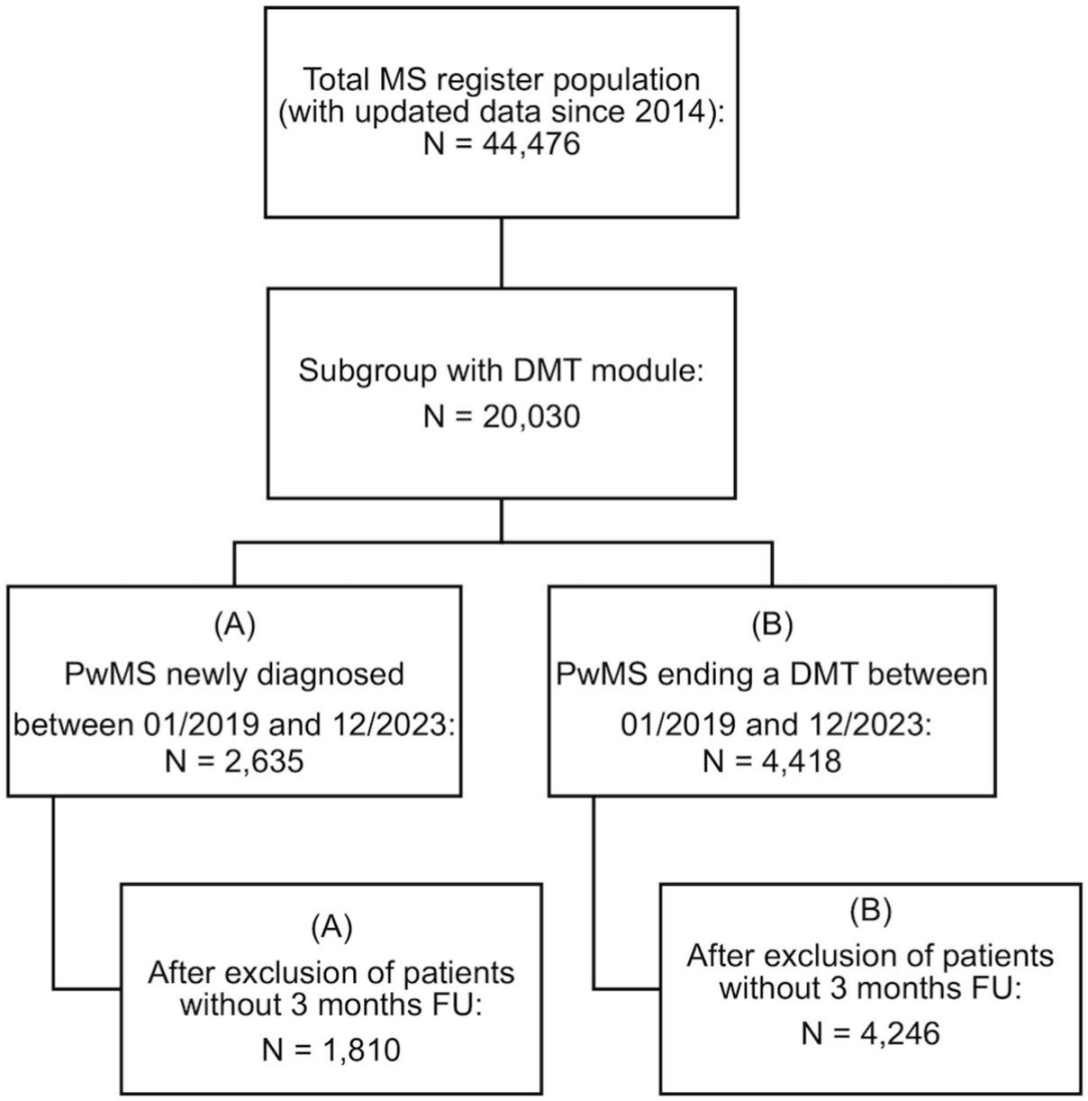
Flowchart illustrating the inclusion criteria and patient selection process. *MS* multiple sclerosis, *DMT* disease-modifying therapies, *PwMS* people with MS, *FU* follow-up

In 2001, the German Multiple Sclerosis Register (GMSR) was established by the German MS Society with the aim of collecting sociodemographic, clinical, and therapeutic data on PwMS and supporting MS research in Germany [35–37]. This multicenter, observational, retrospective study utilizes data from 44,476 PwMS enrolled in the registry since 2014 to provide insights into trends in DMT utilization before, during, and after the COVID-19 pandemic. Inclusion criteria for the study comprised patients with diagnosis of MS, with age ≥ 18 years and complete DMT documentation available. Patients with missing data or inability to provide informed consent to participate in the GMSR were excluded. Two cohorts were retrospectively defined: (A) patients with the date of diagnosis between Jan 2019 and Dec 2023 (*N* = 1810, see Fig. 1) or (B) patients who discontinued or switched DMT between Jan 2019 and Dec 2023 (*N* = 4246). Patients lacking a 3-month follow-up were excluded from the analysis. Treatments were categorized into medium-efficacy DMT (meDMT), including interferons, fumarates, glatiramer acetate, and teriflunomide, and high-efficacy DMT (heDMT), such as natalizumab, alemtuzumab, cladribine, S1P-receptor modulators, and B cell-depleting therapies (BCD).

### Study periods

Four distinct time periods were defined for this analysis: the pre-pandemic period (January 2019–December 2019), the early pandemic period (March 2020–February 2021), the late pandemic period (March 2021–February 2022), and the post-pandemic period (March 2022–December 2023).

### Statistical analysis

Statistical analyses and graphical representations were conducted using R stat 4.3 (R Foundation, Vienna, Austria). Descriptive statistics were applied to both demographic and clinical variables. Continuous variables were summarized as mean and standard deviation (SD), accompanied by 95% confidence intervals (CIs), while categorical variables were reported as frequencies and percentages. *p* values smaller than *α* = 0.05 were considered statistically significant.

## Results

### Demographics and clinical characteristics

During the study period, a total of 44,476 patient visits were recorded, of which, after exclusion of data with less than 3 months follow-up, (A) 1810 individuals were newly diagnosed with MS and (B) 4246 patients discontinued or switched DMT during the pandemic. Demographics and clinical characteristics of the cohort are presented in the Tables 1 and 2.The mean age at diagnosis in cohort A was 36.5 years (range 14–76 years; SD: 11.6) and 68% were females (95%-CI 66.1–70.4%). In cohort B, the average age at DMT stop date was 43.8 years (range 17–81; SD: 12.3), the average disease duration was 12.5 years (range 0–55; SD: 9.2), and 73% were females (95%-CI 71.6–74.3%).

**Table 1:** Cohort A (*n* = 1810); therapy initiation during the pandemic among newly diagnosed pwMS

	Female, % (CI)	Age at diagnosis (mean ± sd)	No therapy, % (CI)	meDMT, % (CI)	heDMT (incl. BCD), % (CI)	BCD , % (CI)
Pre-pandemic (*N*_inc_ = 602; *N*_unc_* = 5)	69.7% [65.9–73.3]	36.0 (11.5)	46.2% [42.1–50.3]	39.2% [35.3–43.2]	14.6% [11.9–17.7]	7.5% [5.5–9.9]
Early pandemic (*N*_inc_ = 472; *N*_unc_* = 8)	68.3% [64.0–72.5]	36.8 (11.4)	47.5% [42.9–52.1]	33.3% [29.0–37.7]	19.3% [15.8–23.1]	9.5% [7.0–12.5]
Late pandemic (*N*_inc_ = 375; *N*_unc_* = 5)	67.9% [62.9–72.6]	35.7 (11.2)	41.9% [36.8–47.0]	29.1% [24.5–33.9]	29.1% [24.5–33.9]	12.8% [9.6–16.6]
Post-pandemic (*N*_inc_ = 341; *N*_unc_* = 2)	66.2% [60.9–71.2]	37.8 (12.2)	37.8% [32.7–43.2]	20.8% [16.6–25.5]	41.3% [36.1–46.8]	28.7% [24.0–33.9]

*CI* confidence interval, *PwMS* people with multiple sclerosis, *meDMT* medium-efficacy DMT, *heDMT* high-efficacy DMT, *BCD* B cell-depleting therapies

*N_unc_ refers to the number of DMT episodes that were unclassifiable, e.g., *study medication*, and thus not considered for percentages

**Table 2:** Cohort B (*N*_episodes_ = 5461, *N*_patients_ = 4246); DMT discontinuation and switch during the pandemic

	Female, % (CI)	Age at DMT stop (mean ± sd)	Disease duration at DMT stop (mean ± sd)	Discontinuation > 3 M, % (CI)	Switch to meDMT, % (CI)	Switch to heDMT, % (CI)	Switch to BCD, % (CI)
Pre-pandemic (*N*_episodes_ = 1745) (*N*_patients_ = 1570)	72.2% [69.9–74.4]	43.2 (12.1)	12.0 (9.1)	47.2% [44.7–49.6]	18.8% [17.0–20.8]	34.0% [31.7–36.3]	16.8% [15.0–18.7]
Early pandemic (*N*_episodes_ = 1228) (*N*_patients_ = 1143)	71.6% [68.9–74.2]	43.8 (12.4)	12.2 (9.0)	46.3% [43.4–49.2]	16.4% [14.3–18.6]	37.3% [34.6–40.2]	14.0% [12.1–16.1]
Late pandemic (*N*_episodes_ = 1173) (*N*_patients_ = 1091)	74.1% [71.4–76.6]	43.3 (12.2)	12.6 (9.3)	39.0% [36.1–42.0]	14.3% [12.3–16.5]	46.6% [43.7–49.6]	17.2% [15.0–19.5]
Post-pandemic (*N*_episodes_ = 1315) (*N*_patients_ = 1108)	74.8% [72.2–77.4]	43.9 (12.2)	12.4 (9.0)	33.7% [31.1–36.4]	18.6% [16.4–20.8]	47.7% [45.0–50.5]	22.8% [20.5–25.3]

Percentages (%) excluding unclassifiable DMT, e.g., *study medication*

*CI* confidence interval, *sd* standard deviation, *3M* 3 months, *PwMS* people with multiple sclerosis, *meDMT* medium-efficacy DMT, *heDMT* high-efficacy DMT, *BCD* B cell-depleting therapies

**Fig. 2:**
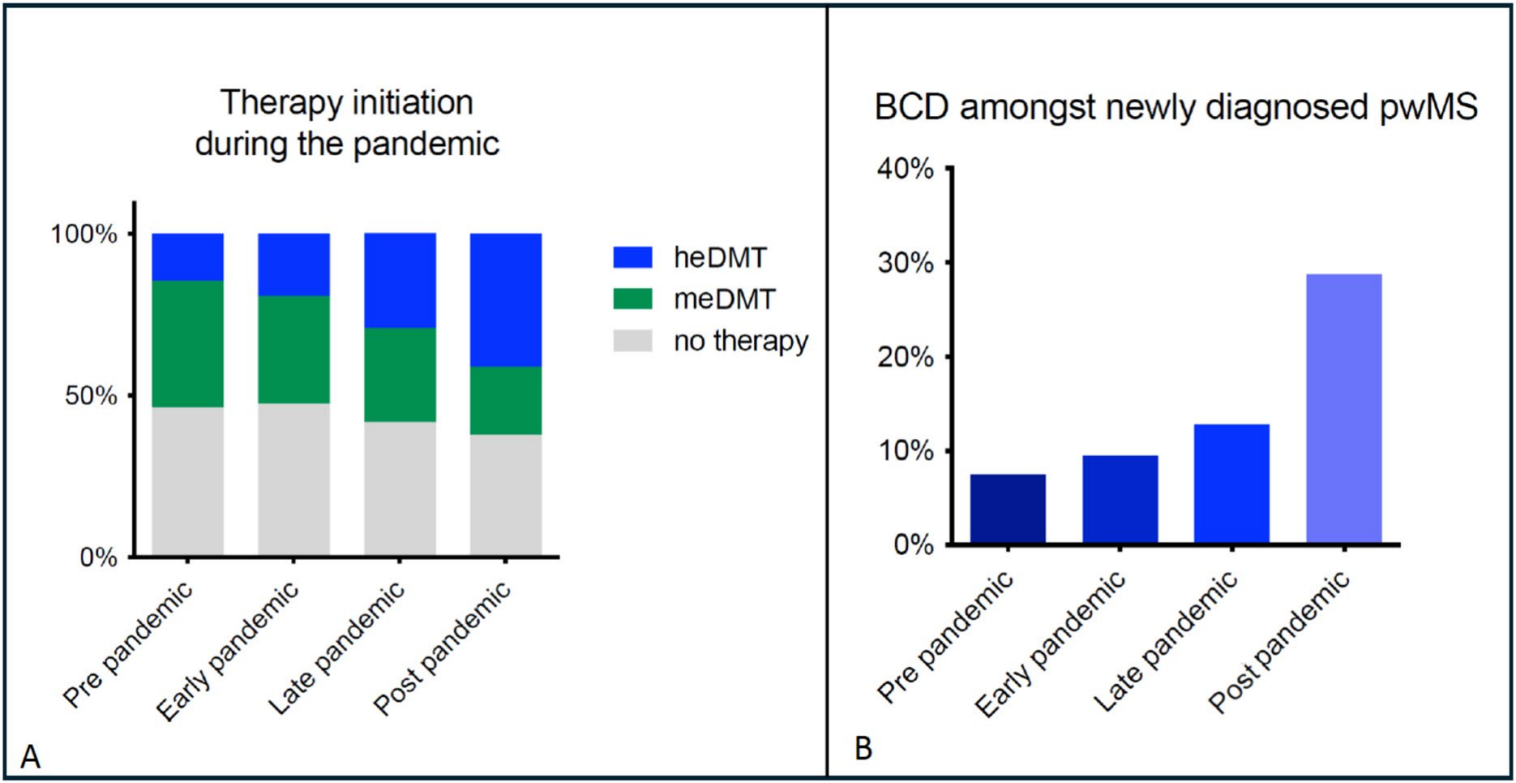
**A** Therapy initiation among newly diagnosed pwMS (Cohort A). **B** B cell-depleting therapy initiation among newly diagnosed pwMS (Cohort A). *PwMS* people with multiple sclerosis, *meDMT* medium-efficacy DMT, *heDMT* high-efficacy DMT, *BCD* B cell-depleting therapies

Among newly diagnosed PwMS (Cohort A; *n* = 1810, Table 1, Fig. 2), 46% in the pre-pandemic period did not initiate a DMT within the first 3 months, while 39% began on a meDMT, and 15% started an heDMT, with 7.5% of them receiving BCD. During the early pandemic, the proportion of patients not early initiating a DMT remained relatively unchanged at 48%. However, this percentage decreased during the late and post-pandemic periods. The proportion of PwMS treated with meDMT gradually decreased during and after the pandemic, from 33% in the early pandemic period to 29% in the late pandemic period, and further to 21% post-pandemic. Conversely, the proportion of patients receiving heDMT increased over time, rising from 19% in the early pandemic to 29% in the late pandemic, and reaching 41% post-pandemic. This shift was particularly notable for BCD, with usage rising significantly from 9.5% in the early pandemic period to 13% in the late period and further surging to 29% post-pandemic (*p* < 0.001).

**Fig. 3:**
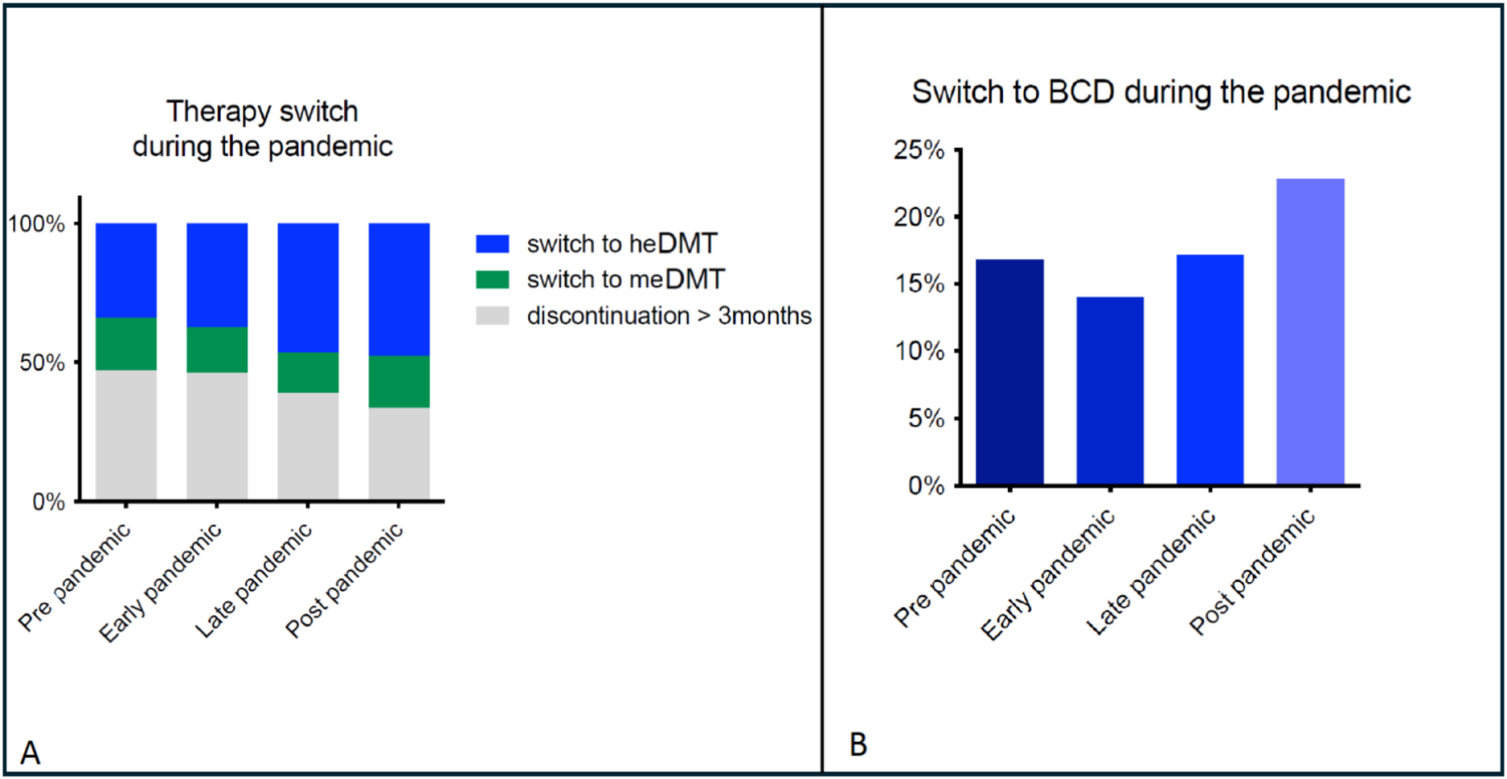
**A** DMT discontinuation and switch during the pandemic (Cohort B). **B** Switch to B cell therapy during the pandemic (Cohort B). *PwMS* people with multiple sclerosis, *meDMT* medium-efficacy DMT, *heDMT* high-efficacy DMT, *BCD* B cell-depleting therapies

Among PwMS who discontinued or switched DMT (Cohort B; *n* = 4246, Table 2, Fig. 3), 47% paused their DMT for ≥ 3 months during the pre-pandemic period, while 19% switched to a meDMT and 34% to a heDMT, with 17% transitioning to BCD. During the early pandemic, the proportion of patients discontinuing DMT for >3 months remained relatively unchanged at 46%, but this percentage decreased in the late and post-pandemic periods to 39% and 34%, respectively. The proportion of patients switching to meDMT remained relatively stable, with a slight decrease in the late pandemic phase (“early” 16%, “late” 14%, “post” 19%). In contrast, there was a notable increase in the proportion of PwMS switching to heDMT over time, rising from 37% in the early pandemic to 47% in the late pandemic, and 48% post-pandemic. This shift was accompanied by a lagged, but marked increase in BCD use, with rates increasing from 14% in the early pandemic to 17% in the late phase, and reaching 23% post-pandemic (*p* < 0.001).

## Discussion

The management of chronic autoimmune conditions like MS, which require long-term therapy, has been particularly challenging for neurologists during the COVID-19 pandemic. While it remains unclear whether PwMS are at increased risk for contracting COVID-19 or experiencing severe outcomes [33], the use of DMTs in MS has been a central concern, with debates surrounding their potential risks during the pandemic [13, 17, 38].

This study used data from the German MS Register to explore how DMT-prescribing patterns evolved during the pandemic. With a total of 44,476 patient analyzed, 6056 patients met the inclusion criteria, comprising 1810 newly diagnosed patients (Cohort A) and 4246 patients who discontinued or switched their DMT (Cohort B). Our findings highlight important shifts in MS care and provide insights into how the global health crisis impacted treatment decisions, offering lessons for future pandemics and healthcare disruptions at both local and global levels.

In contrast to previous studies [19, 39], our findings do not show a significant delay in the initiation of DMTs during the COVID-19 pandemic. Among newly diagnosed PwMS (Cohort A), 46% did not initiate DMT within the first 3 months pre-pandemic, and during the early pandemic, this proportion remained stable at 48%. However, the percentage of patients not initiating a DMT decreased during the late and post-pandemic periods, indicating a shift toward earlier treatment initiation as the pandemic progressed. The initial hesitancy in starting DMTs may reflect uncertainties regarding early treatment decisions and concerns about therapy-related risks and efficacy of vaccination, particularly in the context of COVID-19. This highlights the complexity of managing newly diagnosed PwMS during a global health crisis. For those who did begin treatment during the early pandemic, 33% started on a meDMT, and 19% on an heDMT, with 9.5% opting for BCD. As the pandemic unfolded, a marked shift occurred in treatment patterns, with the proportion of patients starting on meDMT gradually decreasing. By the post-pandemic period, only 21% of newly diagnosed PwMS were receiving meDMT, compared to 33% in the early pandemic phase. In contrast, the use of heDMT increased steadily, reaching 41% in the post-pandemic period. The rise in BCD use was particularly pronounced, tripling from 9.5% in the early pandemic to 29% post-pandemic.

Cohort B, which comprised patients who either discontinued or switched their DMT, also demonstrated notable trends. Prior to the pandemic, 47% of patients had paused their DMT for 3 months or more. In the same period, 19% switched to an meDMT, and 34% switched to an heDMT, with 17% transitioning to BCD. During the early pandemic, the proportion of patients discontinuing DMT remained stable at 46%, but this percentage decreased in the late and post-pandemic periods to 39% and 34%, respectively. The proportion of patients switching to meDMT remained relatively stable throughout the pandemic, with a slight decline during the late phase (16% in the early pandemic, 14% in the late pandemic). However, a significant increase in switching to heDMT was observed, rising from 37% in the early pandemic to 48% post-pandemic. The increase in BCD use was particularly delayed but notable, with usage rising from 14% in the early pandemic to 23% post-pandemic. These findings are consistent with other studies reporting a decline in the use of anti-CD20 monoclonal antibodies during the pandemic [22].

Our results demonstrate a clear shift toward heDMT, particularly BCD, as the COVID-19 pandemic progressed. Neurologists may have initially exercised caution in prescribing anti-CD20 therapies during the early phases of the pandemic due to concerns about their immunosuppressive effects and potential increased vulnerability to infections [9, 40]. Concerns regarding reduced vaccination responses during certain DMTs, particularly with anti-CD20 therapies, were also a significant cause of hesitancy [41]. The long-term impact of COVID-19 vaccination on prescribing patterns for anti-CD20 therapies in MS patients remains an area of active investigation. In general, COVID-19 vaccination has been shown to offer robust protection against severe breakthrough infections in PwMS [42–45]. Emerging evidence indicates however that PwMS treated with anti-CD20 therapies or S1P receptor modulators exhibit a diminished humoral response to COVID-19 vaccines compared to those on other DMTs [46–48] or healthy controls [41]. Recent studies further suggest that the risk of insufficient vaccination response, indicated by lower SARS-CoV-2 antibody levels, persists in PwMS undergoing anti-CD20 treatment, even after repeated exposure to the vaccine or virus [49].

The shift toward heDMT, particularly BCD, during and after the COVID-19 pandemic likely reflects both clinician and patient responses to the unique challenges posed by the pandemic, coupled with growing confidence in the safety of these treatments [50]. The pandemic exacerbated pre-existing challenges in MS management, including treatment discontinuation and difficulties in maintaining regular follow-ups. These disruptions likely influenced prescription patterns, as clinicians aimed to reduce the frequency of therapy adjustments and ensure more consistent disease control during a period of restricted healthcare access. The shift toward more potent therapies in the later pandemic phase may reflect the increased confidence clinicians had in managing patients due to the resumption of regular follow-up appointments and in-person visits. The approval of subcutaneous formulations, such as ofatumumab in March 2021 [51], may have further contributed to the increased preference for BCD therapies in the later stages of the pandemic.

Treatment decisions during this pandemic period required careful consideration of the individual patient's risk-to-benefit ratio, particularly with regard to established risk factors for severe COVID-19 outcomes. Misconceptions about the risks of SARS-CoV-2 infection in MS patients may have contributed to harmful choices, such as avoiding necessary medical visits, delaying DMT start, or discontinuing medications, thereby compromising MS care. In the absence of clear, evidence-based guidelines, neurologists were tasked with balancing the potential risks posed by COVID-19 to immunosuppressed patients against the consequences of delaying or undertreating MS. Delaying treatment, de-escalating therapy, or interrupting DMT regimens in anticipation of vaccine availability could lead to suboptimal disease management and progression [33]. Addressing these challenges necessitates localized and population-specific strategies, including efforts to identify and address knowledge gaps. Patient education, the provision of credible information, and fostering personal responsibility for treatment adherence are critical components of ensuring continuous and effective MS management in the face of future healthcare disruptions.

### Limitations

Our register-based study has several limitations. The patients’ co-morbidities and COVID-19 vaccine status, which could have influenced DMT choice, were only available for a subgroup of patients and thus not used. PwMS included in the registries may not represent all individuals in the respective healthcare system (e.g., differences in DMT prescribing related to health care environment and limitations in access during the COVID-19 pandemic) and MS treatment practices may exhibit variability between centers. In addition, there is potential for selection bias in the registry (e.g., the GMSR recruits PwMS from centers awarded a certificate by the German MS Society after fulfilling certain criteria defined by the Society). Given the unforeseeable nature of a pandemic, this study inherently carries the limitations associated with a retrospective design. It is challenging to determine whether changes in treatment prescriptions are directly attributable to the COVID-19 pandemic or are due to temporal fluctuations inherent in prescriptive patterns that may be affected also by registration of new compounds and routes of administration, and new study data and treatment recommendations.

## Conclusion

This study highlights shifts in DMT-prescribing patterns during and after the COVID-19 pandemic. We observed no significant delays in DMT initiation or hesitation in treatment resumption during the pandemic. However, an increase in the use of heDMT, particularly BCD, was noted, especially during the later and post-pandemic periods. These changes reflect the challenges neurologists faced in managing MS amidst uncertainties about COVID-19 risks. BCD prescriptions initially decreased likely due to concerns regarding vaccine response and COVID-19 disease severity but increased as clinical evidence accumulated, showcasing the adaptability of neurologists in managing MS care. The growing preference for BCD therapies was likely further driven by the approval of subcutaneous formulations, and growing trust with growing personal experiences. Understanding the knowledge, attitudes, and behaviors of MS neurologists will be crucial in preparing for future pandemics.

## Data Availability

Anonymized data will be made available on request for any qualified investigator under the terms of the registry’s usage and access guidelines and subject to the informed consent of the patients.

## Abbreviations

MS: Multiple sclerosis
COVID-19: Coronavirus disease-19
PwMS: People with multiple sclerosis
DMT: Disease-modifying therapies
meDMT: Medium-efficacy DMT
heDMT: High-efficacy DMT
BCD: B cell-depleting therapies
ECTRIMS: European Committee for Treatment and Research in Multiple Sclerosis
mAbs: Monoclonal antibodies
GMSR: German Multiple Sclerosis Register
FU: Follow-up
SD: Standard deviation
CI: Confidence interval
